# RELATIONS BETWEEN MOOD AND DAILY ACTIVITIES: AN ECOLOGICAL MOMENTARY ASSESSMENT

**DOI:** 10.1093/geroni/igad104.3130

**Published:** 2023-12-21

**Authors:** Abel Pichardo, Brent Mausbach

**Affiliations:** San Diego State University, San Diego, California, United States; University of California, San Diego, San Diego, California, United States

## Abstract

Behavioral activation (BA) is an evidence-based therapy for depression that has recently been applied for informal caregivers. Investigating the mechanisms of BA could lead to enhanced quality of care by optimizing guidance to patients. We examined concomitant and lagged associations between mood and daily activities in 50 informal caregivers using an Ecological Momentary Assessment approach via smartphone technology. Participants recorded the number and types of activities they did each day in a 14-day period. Activities were rated for enjoyment on a scale of 1 to 5 (5 being most pleasant). Mood for the day was rated on a scale of 1 to 5, (5 being the most positive). Linear mixed-model analyses examined concomitant and lagged (+/- 1 day) associations between activities and moods. Results indicated concomitant associations between mood and both frequency (t = 2.36, p = .019) and enjoyment of activities (t = 4.11, p < .001). Previous day’s mood was not associated with either frequency or enjoyment of activities the following day. Previous day’s enjoyment of activities was associated with mood the following day (t = 2.34, p = .02). There was no association between frequency of activities the previous day and mood the following day. These findings support BA theory by suggesting that increasing engagement in enjoyable activities may have positive impacts on mood not only in the moment but also have a lasting impact on future mood. Future research should expand this model by including covariates important to the population such as age and activity restriction.

